# Type 2 diabetes patients’ perspectives on lifestyle counselling and weight management in general practice: a qualitative study

**DOI:** 10.1186/1471-2296-15-97

**Published:** 2014-05-15

**Authors:** Matthias Wermeling, Ulrike Thiele-Manjali, Janka Koschack, Gabriele Lucius-Hoene, Wolfgang Himmel

**Affiliations:** 1 Department of General Practice/Family Medicine, University Medical Center Göttingen, Göttingen, Germany; 2 Freiburg Institute for Advanced Studies (FRIAS) and Institute of Psychology, University of Freiburg, Freiburg, Germany

**Keywords:** Type 2 diabetes, Physician-patient relations, Obesity, Eating, Patient education, Family practice, Qualitative research

## Abstract

**Background:**

Lifestyle counselling is a pivotal aspect of diabetes care. But general practitioners (GPs) often have problems in finding their role in patients’ weight management. The aims of this study were to investigate the experiences of type 2 diabetes patients with lifestyle counselling from their GPs and to explore how patients’ preferences regarding counselling are embedded in the context of self-management and wider cultural aspects of nutrition.

**Methods:**

Narrative interviews were conducted with 35 people with type 2 diabetes aged between 35 and 77 years. The interviews were transcribed verbatim and analysed using the thematic framework method.

**Results:**

Many patients had a strong feeling of personal responsibility for weight reduction as integral to diabetes self-management but found it difficult to integrate the changes their disease requires into their self-management activities. They attached great importance to their GPs’ advice on diet. While some patients appreciated direct communication, others regarded dramatic pictures as either unhelpful or offending. A serious problem was the incompatibility of the dietary recommendations with daily life resulting in a reluctance to adjust the whole diet to the needs of diabetes care.

**Conclusions:**

Ambivalence towards patient self-management and tensions between the necessary changes to patients’ lifestyles and their culture, makes the GP’s role difficult and full of conflict. Instead of focusing exclusively on the guidelines of diabetes management and provision of information, GPs should explore the patients’ capabilities of self-management through open communication and accept their patients’ wishes to protect nutrition as part of their culture.

## Background

Type 2 diabetes impacts lifestyle habits such as eating, smoking and physical activity much more than other medical conditions and it requires regular glycaemic monitoring [1]. Excessive eating as well as an inactive lifestyle are, in addition to genetic factors, considered the major causes for type 2 diabetes [2]. Consequently, lifestyle interventions are endorsed as one of the most promising therapeutic options [1,3] and evidence suggests that type 2 diabetes patients would like to receive nutritional advice [4].

Given the possibility of regular contact and the often close relationships patients have with their general practitioner (GP), GPs play a pivotal role in lifestyle counselling and self-management education [5]. While lifestyle counselling in primary care is generally considered to be effective [6], GPs reported having difficulty in finding their role in patients’ weight management [7-9]. At the same time, patients have also reported experiencing a paternalistic attitude [10] or to have been accused of cheating by professionals [11]. Obese diabetic patients, especially women, feel their physicians provoke feelings of being stuck, defiance, guilt or shame [12].

One reason for these difficulties and tensions between patients and physicians may be that communication about eating, weight loss and exercise is not so much a technical problem, but instead infringes on individual autonomy and self-identity [13] as well as cultural and personal aspects of what constitutes healthy food and social eating practices. Therefore, it seems likely that individual preferences and social or cultural values not only shape a patient’s behaviour but also influence their experience of lifestyle counselling, especially dietary advice. The aim of this study was to explore the type 2 diabetes patients’ perception of diet counselling by GPs. Moreover, by adopting a narrative approach towards data collection we wanted to give patients the opportunity to provide rich accounts of their experiences and to embed their perceptions in the wider context of diabetes self-management and cultural aspects of nutrition.

## Methods

### Context and setting

A qualitative design was employed to study patients’ experiences and preferences in detail. The study made use of a large database of patient experiences which provided the basis of the website http://www.krankheitserfahrungen.de. This website is based on the idea and methods of the British website http://www.healthtalkonline.org. It contains interview sequences as video, audio or text about experiences of people suffering from chronic conditions. Both the German and British projects are part of DIPEx International (http://www.dipexinternational.org). The main goal of the DIPEx project is to give patients the opportunity to learn from others and to have access to free information distinct from that provided by medical experts or dubious internet sources. The interview data are also used in healthcare and inter-professional medical education. For more details see Ziebland and Herxheimer [14] and the information on the aforementioned websites.

### Participants

#### Sample

We mainly recruited patients with assistance from primary care practices as they represent a broad variety of patients with diverse illness experiences. Other participants were recruited from self-help groups, local clinics or the wider local community e.g. Islamic centres. It was intended that this sampling strategy would also help ensure that we also included people who may be reluctant to regularly visit a doctor. Participants came from different urban and rural areas in Germany.

To assure a maximum variation of experiences, patients were recruited according to a proper age-sex-mix. After we had recruited about half of the sample, we conducted an interim analysis of the data. For this interim analysis, experts from health care, medical research, self-health groups and other relevant stakeholders were invited to form an advisory board. This advisory board scanned the interviews conducted and tried to find out whether or not relevant patients and/or types of the disease under study were missing. We became aware that we had predominantly recruited older people. As we were very interested to give younger people with type 2 diabetes a voice, we drew a second sample consisting of people in a younger age bracket. And as we also only had a few people with a severe course of the disease and major complications, we similarly considered it very important that people with a severe condition should find discussions from their peers on our website. So we also tried to include more people with severe conditions in the second period of sampling. During the subsequent analysis, saturation was considered achieved. People with severe cognitive impairments were excluded from the sample as it would have been very difficult for them to participate in narrative interviews.

#### Data collection

Interviews and interim analysis took place between 2008 and 2010. Interviews were conducted by qualified interviewers (UT being one of them) either one-to-one in the participant’s home or in the department of the university. In order not to narrow down the experiences of the participants to predefined themes and assumptions, a narrative interview technique provided the basis of the website project. A narrative interview technique allows participants to express their experiences using their own words and the interview is structured according to areas relevant to them. The interview began with a section in which an open question invited the participants to relate their stories from the moment when they first suspected something was wrong with their health. Prompts and probes were used, when appropriate, to elicit further information. When the initial story was finished, a few follow-up questions were asked referring to, amongst other items, the doctor-patient-relationship and sources of support in diabetes care. All interviews were digitally recorded (32 interviews were videotaped and 3 interviews were audiotaped) and transcribed verbatim.

### Analysis

While only small sections of the interviews are published on the website, we used the original narrative interviews for this study. The interviews were analysed according to the ‘framework’ approach [15]. This approach was originally developed for policy relevant studies but it is now widely used in qualitative health care research because it allows deductive and inductive research questions equally [16]. The procedure of analysis includes five steps: familiarisation, identifcation of a thematic framework, indexing, charting, mapping and interpretation. In the context of our study, this meant that first the interviews were read several times and discussed by members of the research group. The issue of weight and experiences with dietary advice from GPs was identified as one dominant pattern in the data that looked promising for further investigation. Next, one of the authors (MW) developed a coding scheme and coded the transcribed interviews until saturation was reached, which meant that no further codes or categories could be found in the data. Initial coding was supported by the data analysis software ATLAS.ti, v5.1. We then charted the data according to the emerging framework of key themes in order to compare across the different cases. Analysis finally involved refining emerging concepts and finding associations between and within the interviews.

Rigor was ensured with continuing discussions during data collection and analysis within the research team. Moreover, two of the authors (UT and WH) coded large parts of the data in order to check the coding scheme and the emerging themes. In case of disagreement, codes and results were discussed until a consensus was reached.

### Ethics

All participants gave prior written consent. The study was approved by the local ethics committee (University of Göttingen, Medical Faculty, no. 18/1/09).

## Results

The total sample consisted of 35 participants (16 female, 19 male) aged between 35 and 77 years (mean: 59 years). The interviews lasted, on average, 104 (range: 43–220) minutes.

Nearly all patients talked about weight and diet in the interviews, most of them in a very detailed way. Their narratives about weight and diet could be placed into three interrelated categories: (1) difficulties with self-management and the role of the GP, (2) preferred styles of lifestyle counselling, and (3) dietary recommendations in a cultural context.

### Difficulties in self-management and the role of the GP

The majority of participants were aware of the close connection between overweight and unhealthy eating. Many considered weight the cause of their condition. A strong feeling of personal responsibility was expressed in their narratives and weight reduction often was regarded as an integral part of diabetes self-management. Independent of their GPs’ advice or admonition, patients often reported the need for discipline in order to reduce weight.

Actually it was always my ambition to lose weight. Well…it concerns me and yes, of course, I would prefer to have a normal weight rather than being overweight […]. There are only disadvantages - no advantages whatsoever. Expensive, large sized clothes and impairments to health - these are not advantageous (ID 38, male).

Sometimes, their impulse for dieting developed over time and required self-monitoring. In a few cases an effort to reduce weight was not even assisted by the GP.

And I say to myself: I waited and saw what happened over the last couple of months, but honestly, I am going to lose weight eventually. This does not come from my GP as he says: “Everything is fine and within reason”. I have to do something (ID 32, male).

But many patients reported having problems with self-management and therefore attached great importance to their GPs’ comments and advice on weight and diet. However, for these patients the nature of their disease did not prompt them to make changes. Patients remarked that an absence of pain and discomfort due to diabetes was a major obstacle to self-management and an adjustment to their lifestyle via a rigorous diet. They did not feel “real” pain as such, nor experienced any real limitations that reminded them of their illness.

Some participants indicated that they underestimated the potential consequences of diabetes and found it difficult to constantly keep track of weight and blood sugar levels. Although these patients stated that they were aware of the importance of weight and blood sugar levels, they found it difficult to adapt their behaviour to reflect this awareness. In spite of their fierce plea for self-management and self-responsibility, they often relied on their GP to bridge the gap between their understanding of the consequences and their motivation to take concrete action. This was frequently expressed as a desire to receive a wake-up call.

If need be, he has to give me a wake-up call now and again and then I feel better afterwards and take care over my consumption of carbohydrates. That works wonders. We complement each other well (ID 34, female).

When this happens most respondents still took responsibility for weight related symptoms and valued the rebuke from their GP. The difficulty in self-control and self-management often made itself felt in terms of guilt, shame and misbehaviour.

And when my sugar levels are quite high once more for a longer period of time I call the GP for an appointment and then I get my direction from her yet again. She is outspoken, you know; she doesn’t hold back. I do appreciate that, you know. It is my own fault anyway (ID 36, male).

### Preferred styles of lifestyle counselling

Although nearly all patients mentioned that they were referred to external nutrition counselling or to a rehabilitation facility, they reported that the topic of diet and weight was frequently discussed with their GPs. Apart from some people who did not necessarily expect dietary advice when consulting their GP, the patients could be roughly divided into two categories concerning the words their GPs should use in conversation about weight and weight reduction. While some patients appreciated a rather drastic form of communication, others regarded dramatic pictures as either unhelpful or even offending. In particular, those patients who insisted on self-responsibility but had difficulties in controlling body weight, valued clear words when it came to weight reduction. According to such patients, the GPs’ words sometimes were seen as a ‘turning point’ and resulted in stricter weight management.

Then he gave me a fierce telling-off; bit my head off, really. And I thought “Yes I know. I know it all”. I was a nervous wreck and this was the decisive point for me to say “Yes - and now you do it [lose weight]” (ID 40, female).

I am very happy with that GP and as I said he is straight-forward and tells you everything (ID 36, male).

For many patients concrete advice and a trusting relationship were the pre-condition for this style of speech. Patients who had a long-lasting relationship with their GP appreciated when he or she was forthright about the necessity of weight reduction.

The GP would tell me straightaway that “this is not on, my friend”. I like this GP a lot as I have the opinion that I can talk openly to him about everything, about my problems and so forth; that would not happen if I did not trust the doctor. Therefore I do understand when he is straight forward, as I know he means well (ID 36, male).

In contrast to the above, other patients criticised the physicians’ instruction to reduce weight as a way to lay responsibility for a successful diabetes management with the patient. Patients in particular who previously indicated having trouble with self-management reported the need for detailed information on diet. For this group, the simple request to reduce weight was useless except when it was accompanied by concrete advice on how to achieve this.

The GP really fired words at me, but I couldn’t care less. All I can say is that this is a standard issue during all the conversations within a medical environment. Everyone seems to push one thing or another and everyone seems to agree that something can be achieved. Sure, what else can they do […] and it is quite handy, of course, to say as soon as you are in the door “first of all you have to lose 10 or 15 kilos and then you will feel much better” (ID 31, male).

Patients also expected their doctor to acknowledge their difficult situation and their endeavours. Patients emphasised that their dietary habits are rooted in their biographical context and have developed over many years. For them, change was a long process even when agreed that it was necessary to prevent co-morbidities associated with diabetes.

You cannot tell someone who has done the wrong thing for years: “If you lose weight it will change”. It may be correct and you know it yourself anyway. But you have to bide your time, you have to talk and apply first aid (ID 49, female).

According to some patients, the very use of words concerning weight in the conversation was crucial. As the following statement suggests, the orthopaedic surgeon’s choice of words and his disrespect made this woman feel offended. She thinks a more moderate style would make it easier for her to appreciate that her body weight is a problem.

The orthopaedic surgeon did not know me at all and he came in, no “hello”, no nothing; just turned around saying across his shoulder: “Have you always been that fat?” The word “fat” made me jump up of the couch fast. To this day I still do not know how I did it. And then I gave him a roasting which probably could be heard 3 rooms away. It would have been different if he had asked “Have you always been that size?” (ID 27, female).

### Dietary recommendations in the cultural context

Although the majority of diabetic patients appreciated the doctors’ attentiveness regarding problems of diet and weight, the most serious problem according to our participants, was the incompatibility of the dietary recommendations with daily life and their views of eating culture. In this context, the topic of eating culture refers to the social aspects of cooking and eating, the pleasure of a tasty meal and the relationship between food and identity. Again, it was primarily those patients with difficulties in finding a suitable diet on their own who reported that the advice from GPs and other health professionals about diet was persuasive in theory but unrealistic in practice. Back at home the information was not capturing their eating habits or the environment in which they lived.

Okay, I do not really adhere to it [the diet] as I am a chef by trade. I prefer to cook with butter and cream and it is not easy to change completely (ID 43, male).

I cook what they [the children] like most. That’s not necessarily healthy. But to cook two dishes – that seems stupid (ID 60, female).

Some patients more fundamentally criticised the philosophy behind the medical approach to diet that provided the basis for the GPs’ lifestyle counselling and nutritional training.

Eating is a matter of culture and I am very much interested in culture - just kidding a little bit. It is not just about the intake of food. […] I must have a sort of a blockage somewhere. If I hear at the back of my mind: “Yes, you have to”. Yes, I do have to lose weight, of course. […] Um, well, the requirements to do this may not have been accepted yet by my subconscious mind (ID 41, male).

The patients’ reluctance to change their whole diet to accommodate the requirements of diabetes care was present in many narratives. For example, patients did report deviating from a diabetes-adjusted diet sometimes. An individual’s attempt to enjoy life overruled the strict instructions regarding diet. As the following quotation suggests, this could mean protecting those things that give meaning to life.

A few sins here and there must be allowed. Otherwise life would have no meaning whatsoever (ID 33, male).

By and large, patients regarded themselves as compliant to the recommendations of their GPs. For them it was a matter of principle rather than a lack of knowledge since they were aware that a strict diet would be better for them given their condition. In this regard, it would appear that opting out of strict eating regimes becomes a sign of autonomy and therefore is a part of the individual identity.

But I said that I am going to eat bread and dripping once a month – it has to be done (ID 57, female).

Some patients indicated that such deviation from a strict diet was allowed by their GPs. In such cases, they often assumed that their GPs accepted a lack of discipline now and then as long as it was only limited to a few situations.

## Discussion

A GP’s role in diabetes management is difficult and full of conflict given the ambiguities in patient self-management and the tensions between required lifestyle changes from the current lifestyle, especially with respect to food culture. The patients’ self-management capabilities and different experiences of how to integrate dietary recommendations into daily life framed their perception and assessment of dietary counselling and determined their preferences concerning the style of communication from their GPs. Although a majority of participants considered weight the cause of their condition, they felt that strict diabetes management sometimes endangered their quality of life. Therefore, they insisted that some pleasures were necessary to maintain their well-being.

Research has shown that people with type 2 diabetes who regard an unhealthy lifestyle as a cause for their condition were more willing to take responsibility and to respond with diet and exercise than those who emphasised genetic factors [17]. Accordingly, most patients in our study regarded their individual lifestyle both as a major cause of their diabetes and an important factor in disease management. At the same time, our interviews revealed ambivalence for self-management. While most patients were aware of the important role of weight and diet in diabetes management, they also insisted on retaining eating habits they deemed crucial in maintaining well-being. This problem of navigating between discipline, culture and quality of life is not just an individual matter, but pervades the doctor-patient-relationship and therefore understanding this may help to better understand when and why GPs play such different roles for patients.

The lack of severe near-term consequences may provide an explanation for the positive role of the GP and other health professionals in diabetes care. Patients usually regard type 2 diabetes as not so serious than did healthcare professionals [18]. This is in line with the narratives of many participants in our study who also did not perceive their condition as painful or dangerous. Consequently they highly valued their doctor as someone who constantly points out the risks of type 2 diabetes. Furthermore, some of these patients either accepted or even favoured a more direct or aggressive style to address their failure with regard to diet and exercise. Doctors that highlighted this helped them to wake up and to remember the need for treatment compliance. While a Swedish study [12] in which diabetic patients, especially obese women, reported feeling humiliated and ignored when health communication was technical and without individual support, we would instead suggest a more complex view. The perception of lifestyle counselling seems to be not only a matter of communication, but it also relates to a patient’s personal self-management capabilities and his or her resources. As an ethnographic study [19] has shown, these different individual backgrounds highly influence patient self-management behaviour. However, our results would indicate that a patient’s perception of the encounter is also relevant. In consequence, a rough communication style may not necessarily be perceived as offending but may be perfectly appropriate for some patients’ needs.

With regard to the strong discipline that is normally required to maintain a diabetes adjusted diet, patients in our sample drew a line beyond which they deviated from their GPs’ recommendation. They insisted on some pleasures that were formed and reinforced by the manifold variations in their backgrounds and therefore non-negotiable in lifestyle counselling. While empowerment studies recommend GPs to accept their patients’ priorities [20,21], this might be a challenge. Not only are patient priorities and preferences sometimes in conflict with the guideline recommendations of diabetes management, but doctors, even those trying to avoid a paternalistic attitude, may feel uncomfortable when confronted with detrimental patient behaviour. As a study on hypertension [22] suggests, it might be necessary in this situation to give up conceptualising and assessing patient behaviour within a strict dichotomy of compliance and non-compliance. Instead, GPs should provide the time, privacy and patience to enable their patients to accept the diagnosis and to find their own way of managing their disease.

Many of our narratives can be viewed as documents indicating an ambivalent doctor-patient relationship. Superficially, this would seem to reflect difficulties integrating dietary advice, while useful and accepted by the patient, into everyday life. But a closer look at the narratives disclose a deeper conflict that seems to be rooted in the discrepancy between the world of medicine and the “lifeworld” of patients as Mishler [23] puts it. When doctors, following evidence-based guidelines for the management of diabetes, opt for a strict diet they will inevitably invade the patient’s world. Patients may have the impression that not only their eating habits but also their entire culture will be taken over by medicine. Not surprisingly, patients may feel the impulse to protect their identities [13]. This kind of control over their own lives was apparent when the participants spoke about the limits of nutritional counselling. They appreciated when their conception of nutrition as a part of culture and integral component of well-being was shared and backed by their GPs, and included an occassional deviation from a strict diet. But some patients made clear in nearly a rebellious tone that no one should try to forbid them this occasional pleasure.

### Strengths and limitations of the study

The open nature of narrative interviews allows the interviewee to speak about those things they regard as relevant. In most interviews the topic of weight and diet was addressed without a direct question from the interviewer. This appears to confirm the impression that weight and diet are of utmost importance to both the patients and the researchers. The role of primary care providers was often voluntarily addressed, too, emphasising the importance of these professionals and their expert dietary advice for diabetic patients.

There are nevertheless potential limitations to this study that should be noted. First, although narrative interviews are considered grounded in people’s real lives [24], caution is recommended in the case of weight and diet. These issues receive significant attention in western cultures and our data may be influenced by the participants’ attempts to present themselves as disciplined and adherent. Therefore, answers might be prone to issues of social desirability. Second, the GPs’ assistance in recruiting participants might have resulted in selection bias because GPs could make a preliminary identification of patients. We tried to avoid this by asking different GPs from various areas. Third, three of 35 interviews were audiotaped instead of videotaped. This might lead to bias because there is no access to social cues if not videotaped. Finally, as with most results of qualitative research, the findings of the study presented here are exploratory and should be considered hypotheses.

## Conclusions

Our findings suggest that patients’ views on illness causation, their self-management capabilities and the different experiences of how to integrate dietary recommendations into daily life have a strong influence on their perception of diet counselling from their GPs. Although patients acknowledged the role of weight and eating in the aetiology of type 2 diabetes, they found it difficult to put this knowledge into practice. Dietary counselling therefore seems to be much more than the rational exchange of information. As a consequence, GPs should explore their patients’ capabilities of self-management in open communication and accept their wish to protect nutrition as part of their culture. In the end they can only acknowledge that the control of diabetes management rests with the patient. However, since individual preferences strongly differ, future research should help doctors to find to a communication style that is aware of the needs of different patients and the pitfalls of addressing control and personal responsibility. Here, promising approaches such as motivational interviewing are well established and should be further implemented in primary care.

## Competing interests

The authors declare that they have no competing interests.

## Authors’ contributions

MW analysed the data and wrote the manuscript. UTM conducted most of the interviews and contributed to analysis and discussion of findings. JK, GLH and WH designed the study and contributed to analysis and discussion of findings. WH supervised the manuscript. All authors read and approved the final manuscript.

## Pre-publication history

The pre-publication history for this paper can be accessed here:

http://www.biomedcentral.com/1471-2296/15/97/prepub
